# Notch-activated mesenchymal stromal/stem cells enhance the protective effect against acetaminophen-induced acute liver injury by activating AMPK/SIRT1 pathway

**DOI:** 10.1186/s13287-022-02999-6

**Published:** 2022-07-16

**Authors:** Mengxue Yu, Min Zhou, Jiahui Li, Ruobin Zong, Yufei Yan, Liangyi Kong, Qiang Zhu, Changyong Li

**Affiliations:** 1 Department of Physiology, School of Basic Medical Sciences, Wuhan University, Wuhan, 430071 China; 2 Department of Biological Repositories, Zhongnan Hospital of Wuhan University, Wuhan, China; 3 Neurocritical Care Unit, The First Affiliated Hospital of USTC, Division of Life Sciences and Medicine, University of Science and Technology of China, Hefei, China; 4 Department of Anatomy and Histology Embryology, Jinzhou Medical University, Jinzhou, China; 5 Children’s Hospital of Nanjing Medical University, Nanjing, China

**Keywords:** Mesenchymal stromal/stem cell, Notch signaling, SIRT1, XBP1, Acute liver injury

## Abstract

**Background:**

Notch signaling plays important roles in regulating innate immunity. However, little is known about the role of Notch in mesenchymal stromal/stem cell (MSC)-mediated immunomodulation during liver inflammatory response.

**Methods:**

Notch activation in human umbilical cord-derived MSCs was performed by a tissue culture plate coated with Notch ligand, recombinant human Jagged1 (JAG1). Mice were given intravenous injection of Notch-activated MSCs after acetaminophen (APAP)-induced acute liver injury. Liver tissues were collected and analyzed by histology and immunohistochemistry.

**Results:**

MSC administration reduced APAP-induced hepatocellular damage, as manifested by decreased serum ALT levels, intrahepatic macrophage/neutrophil infiltration, hepatocellular apoptosis and proinflammatory mediators. The anti-inflammatory activity and therapeutic effects of MSCs were greatly enhanced by Notch activation via its ligand JAG1. However, Notch2 disruption in MSCs markedly diminished the protective effect of MSCs against APAP-induced acute liver injury, even in the presence of JAG1 pretreatment. Strikingly, Notch-activated MSCs promoted AMP-activated protein kinase (AMPKα) phosphorylation, increased the sirtuins 1 (SIRT1) deacetylase expression, but downregulated spliced X-box-binding protein 1 (XBP1s) expression and consequently reduced NLR family pyrin domain-containing 3 (NLRP3) inflammasome activation. Furthermore, SIRT1 disruption or XBP1s overexpression in macrophages exacerbated APAP-triggered liver inflammation and augmented NLRP3/caspase-1 activity in MSC-administrated mice. Mechanistic studies further demonstrated that JAG1-pretreated MSCs activated Notch2/COX2/PGE2 signaling, which in turn induced macrophage AMPK/SIRT1 activation, leading to XBP1s deacetylation and inhibition of NLRP3 activity.

**Conclusions:**

Activation of Notch2 is required for the ability of MSCs to reduce the severity of APAP-induced liver damage in mice. Our findings underscore a novel molecular insights into MSCs-mediated immunomodulation by activating Notch2/COX2/AMPK/SIRT1 pathway and thus provide a new strategy for the treatment of liver inflammatory diseases.

**Supplementary Information:**

The online version contains supplementary material available at 10.1186/s13287-022-02999-6.

## Introduction

Acetaminophen (APAP) overdose is a major cause of acute liver failure worldwide [1]. Although APAP hepatotoxicity is generally thought to be caused by direct interaction of its reactive metabolites with cellular macromolecules, multiple lines of evidence indicates that non-parenchymal cells, especially hepatic macrophages, may also contribute to liver injury indirectly through the release of cytotoxic mediators [2, 3]. Indeed, innate immunity is a major contributing factor in which liver-resident macrophages (Kupffer cells) and recruited monocyte-derived macrophages play a central role in disease progression. Recent evidence indicated that macrophage depletion by gadolinium chloride pretreatment abrogated disease development, whereas their reconstitution by macrophage transplantation restored the sensitivity to APAP [4]. Therefore, evidence from mouse models and early clinical studies supports the notion that targeting pathogenic macrophages can be successfully translated into novel therapeutic strategies for inflammatory liver diseases including APAP-induced liver injury [5].

Multipotent mesenchymal stromal/stem cells (MSCs) exert considerable promise for use in tissue engineering and regenerative medicine as a source of tissue-specific cells owing to unique properties, such as enhanced proliferation, multilineage differentiation and their immunomodulatory properties. With the ability to regulate both the innate and adaptive immune systems, MSCs have great potential for treating various inflammatory diseases in animal models and humans, including acute and chronic liver diseases [6, 7]. The mechanisms by which MSCs exert their therapeutic effects are multifaceted, and most effector mechanisms were shown by the secretion of soluble factors, including indoleamine 2, 3-dioxygenase, nitric oxide, prostaglandin E2 (PGE2), tumor necrosis factor-inducible gene 6, transforming growth factor β and hepatocyte growth factor [8]. Our previous study also demonstrated that MSC-secreted PGE2 contributed to MSCs-mediated immune regulation through a direct interaction between yes-associated protein (YAP) and β-catenin [9]. Although much progress has been made in the use of MSCs to treat various diseases, further insights into the interaction between MSCs and the inflammatory microenvironment will enable us to make better use and enhance the therapeutic effects of MSCs for the treatment of inflammatory diseases.

Various signaling pathways have been implicated in the regulation of MSC maintenance and expansion, including the Notch signaling pathway [10]. Notch signaling is an evolutionarily conserved pathway that regulates multiple biological functions in mammalian, such as cell proliferation, differentiation and fate determination [11]. The Notch family of transmembrane receptors (Notch1-4) determines cell fate through ligand binding and γ-secretase-mediated cleavage that generates Notch intracellular domain (NICD), which binds RBP-J (also named CSL or CBF1) and the nuclear effector Mastermind-like to activate transcription of canonical Notch targets including the hairy/enhancer of split (Hes) and Hes-related family genes [12]. Canonical Notch signaling is well recognized as a regulator of stem cell proliferation, differentiation and self-renewal in the hematopoietic, neural, intestinal, skeletal muscle and bone marrow stromal cells [10, 13–17]. Emerging evidence demonstrates that the Notch signaling pathway has been implicated in regulating innate and adaptive immune homeostasis and function [18]. Recently, data from other groups as well as our own indicated that the Notch signaling modulates liver inflammatory response through multiple mechanisms [19–21]. Intriguingly, a previous report showed that the Notch signaling was required for production of PGE2 by human bone marrow MSCs [22]. This finding suggests that the Notch signaling may be involved in MSCs-mediated immunomodulation. However, it remains largely unknown as to whether and how MSC-specific Notch signaling may influence inflammasome activation and mediate the immunosuppressive effect of MSCs in APAP-induced liver injury.

In the present study, we investigated the therapeutic potential of human umbilical cord-derived MSCs in APAP-induced acute liver injury and identified a previously unrecognized regulatory mechanism of Notch signaling in MSCs-mediated immunomodulation. We demonstrated that Notch activation in MSCs by its ligand Jagged1 (JAG1) increased cyclooxygenase-2 (COX2)/PGE2 production, which in turn activated the AMP-activated protein kinase (AMPKα)/sirtuins 1 (SIRT1) pathway in macrophages, resulting in deacetylation of spliced X-box-binding protein 1 (XBP1s) and subsequent inhibition of NLR family pyrin domain-containing 3 (NLRP3) inflammasome during APAP-induced acute liver injury.

## Materials and methods

### Animal treatment

Male C57BL/6 mice (6–8 weeks old, 17–20 g) were purchased from Hubei Provincial Center for Disease Control and Prevention (Wuhan, China). All animals were housed in sterile cages with free access to food and water at 12 h light/dark cycles. Animal welfare and experimental procedures were performed according to the *Guide for the Care and Use of Laboratory Animals* published by the National Institutes of Health. All animal experiments and protocols were approved by the Committee of Animal Care and Use of Hubei Provincial Center for Food and Drug Safety Evaluation and Animal Experiment.

APAP (Sigma-Aldrich, St. Louis, MO) solution was freshly prepared for each experiment by dissolving APAP in saline at a concentration of 10 mg/ml and warmed to 40 °C. Mice were fasted for 16 h and then either given saline (i.p.) or APAP (400 mg/kg, i.p.). In some experiments, mice were injected via the tail vein with 1 × 10^6^ MSCs (suspended in 200 μl PBS/mouse) or normal human dermal fibroblasts (ATCC, Manassas, VA) 24 h prior to APAP injection. Some mice were injected via the tail vein with bone marrow-derived macrophages (BMDMs, 5 × 10^6^ cells/mouse) transfected with lentivirus expressing XBP1s (Lv-XBP1s) or GFP control (Lv-GFP) 24 h prior to APAP injection. Some mice were injected via the tail vein with SIRT1 siRNA or non-specific (control) siRNA, (2 mg/kg) (Ribobio, Shanghai, China) mixed with mannose-conjugated polymers (Polyplus transfection™, Illkirch, France) at a ratio according to the manufacturer’s instructions 4 h prior to APAP injection as described [23]. Mice were killed for collecting serum and liver tissues at 24 h after APAP injection. Animal survival was observed every 4 h for 72 h until they became moribund.

### Preparation of human umbilical cord-derived MSCs

Human umbilical cord-derived MSCs were obtained from Wuhan Hamilton Biotechnology Co. Ltd. The use of human umbilical cords from healthy donors who gave birth and signed informed consent in Renmin Hospital was supported by the Institutional Ethics Review Board of Renmin Hospital of Wuhan University (Permit Number: WDRY2019-G001). MSCs were isolated, cultured and identified as described in our previous study [24]. MSCs at passage 5 were used in this animal experiment.

### Recombinant JAG1-coated culture plates

Culture plates were coated with anti-human IgG (10 μg/ml) (Sigma-Aldrich) in PBS at 4 °C overnight and then incubated in a solution containing human recombinant JAG1 protein (10 μg/ml, Enzo Life Sciences, Farmingdale, NY) at 4 °C overnight. The same concentrations of human IgG were used to coat plates as controls.

### Lentiviral vector construction

The 293 T cell line was cotransfected by lentivirus packaging vectors with constructed XBP1s-overexpressing lentivirus (p-Lv-XBP1s, Applied Biological Materials Inc., Canada). Cells were seeded in six-well plates and transfected when they reached 60–70% confluence. Cells were cotransfected with p-Lv-XBP1s, psPAX2 and pVSVG using Lipofectamine 3000 reagent (Invitrogen) to package the lentiviruses according to the manufacturer’s instructions, as previously described [21]. Forty-eight hours after transfection, the viral vector-containing supernatant was collected and filtered through a 0.45 μm filter. GFP lentivirus (Lv-GFP, Applied Biological Materials Inc.) was used as a control.

### Hepatocellular function assay

Serum alanine aminotransferase (sALT) levels, an indicator of hepatocellular injury, were measured by an automated chemical analyzer (Olympus Automated Chemistry Analyzer AU5400, Tokyo, Japan).

### Histology and immunohistochemistry staining

Liver sections were stained with hematoxylin and eosin (H&E). The necrotic area was assessed by analyzing at least 10 randomly selected areas per sample, with computer-assisted image analysis with MetaMorph software (Universal Imaging Corporation, Downingtown, PA). Liver macrophages and neutrophils were detected using primary rat anti-mouse F4/80^+^ mAb (Mac-1, M1/70; BD Biosciences, San Jose, CA) and Ly6G^+^ mAb (BD Biosciences, San Diego, CA), respectively. After incubation with secondary biotinylated goat anti-rat IgG (Vector, Burlingame, CA), followed by treatment with immunoperoxidase (ABC Kit, Vector), the average number of positive cells was quantified by analyzing at least 10 random high-power fields (HPF, original magnification × 400) per animal, with Image-Pro Plus software.

### TUNEL staining

The apoptosis of hepatocytes was measured by using a TUNEL Apoptosis Assay Kit (Roche-Boehringer Mannheim, Germany). The TUNEL-positive cells were visually identified by fluorescence microscopy. The results were scored semiquantitatively by averaging the number of apoptotic cells/HPF. Ten fields were evaluated per sample.

### Quantitative RT-PCR analysis

Total RNA was purified from liver tissues or cell cultures using the RNeasy Mini Kit (Qiagen, Chatsworth, CA) according to the manufacturer’s instructions. Complementary DNA synthesis reactions from the RNA (0.5 μg) reverse transcription were carried out using PrimeScript™ RT reagent kit with gDNA Eraser (Takara Biotechnology Co. Ltd, Japan). Quantitative RT-PCR was performed using Hieff™ qPCR SYBR^®^ Green Master Mix (YEASEN) and CFX 96 Detection System (Bio-Rad, Hercules, CA). Primer sequences used for the amplification of Notch1-4, COX2, TNF-α, IL-1β, MCP-1, CXCL-1 and GAPDH are shown in Additional file 1: Table 1.

### Immunoprecipitation

BMDMs were lysed in RIPA lysis buffer containing protease inhibitors. The lysates were incubated with antibodies against IgG (1:1,000, Abcam, MA), acetyl-lysine (1:300, Abcam, MA) and protein A/G beads at 4 °C overnight. After immunoprecipitation, the immunocomplexes were washed with lysis buffer three times and analyzed by standard immunoblot procedures.

### Western blot analysis

Protein was extracted from liver tissues or cell cultures. Monoclonal rabbit anti-mouse Notch2, COX2, phos-AMPKα, AMPKα, SIRT1, XBP1s, NLRP3, ASC, C-caspase-1 and β-actin Abs (1:1,000, Cell Signaling Technology, San Diego, CA) were used. The relative quantities of proteins were determined by a densitometer, and the results were expressed in absorbance units (AU).

### *BMDM isolation and *in vitro* transfection*

BMDMs were generated as previously described [9]. In brief, bone marrow cells were removed from the femurs and tibias of mice and cultured in DMEM supplemented with 10% FBS and 20% L929-conditioned medium. Cells (1 × 10^6^/well) were cultured for 7 days and then transfected with 100 nM of SIRT1 siRNA (Santa Cruz Biotechnology) using Lipofectamine 2000 reagent (Thermo Fish Scientific, Carlsbad, CA). The non-specific (NS) siRNA as controls. In some experiments, BMDMs were transfected with Lv-XBP1s or Lv-GFP control vector. After 48 h, cells were supplemented with 100 ng/ml of LPS for additional 6 h. Then, these cells were harvested and cell lysates were analyzed by Western blot analysis.

### ELISA assay

PGE2, IL-18 and IL-1β levels in serum were measured by ELISA, according to the manufacturer’s standard protocols (eBioscience, San Diego, CA). Absorbance was read on a Multiscan FC plate reader and analyzed with SkanIt for Multiskan FC software (Thermo Scientific, Schwerte, Germany).

### Statistical analysis

Data are expressed as mean ± SD and analyzed by Student’s t tests. Per comparison two-sided *p* values less than 0.05 were considered statistically significant. Multiple group comparisons were performed using one-way ANOVA with a post hoc test. All statistical analysis was performed using SPSS17.0 software.

## Results

### Administration with Notch-activated MSCs enhances the protective effects against APAP-induced hepatotoxicity

**Fig. 1:**
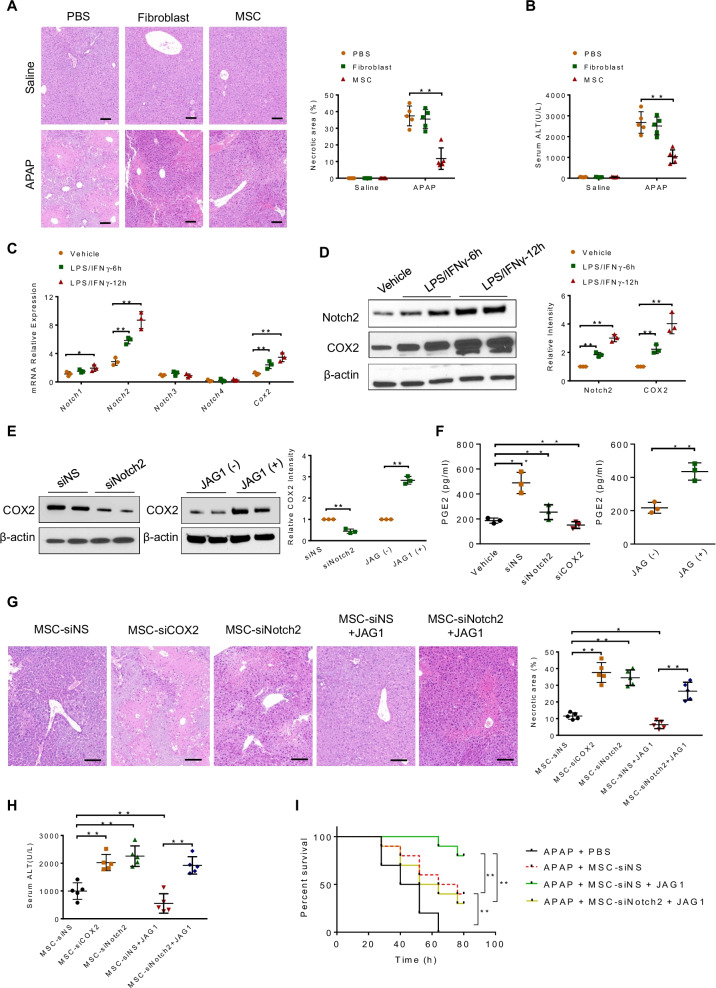
Notch-activated MSCs enhance the protective effects against APAP-induced hepatotoxicity. **A** Mice were subjected to APAP-challenged liver injury. Some mice were injected via the tail vein with human umbilical cord-derived MSCs, fibroblasts (1 × 10^6^) or PBS 24 h prior to APAP injection (400 mg/kg, i.p.). Representative histological staining (H&E) of liver tissue (*n* = 5 mice/group) and percent of necrotic area. Scale bars: 100 μm. **B** Hepatocellular function, as assessed by serum ALT levels (IU/L) (*n* = 5 mice/group). **C** Quantitative RT-PCR-assisted detection of Notch1, Notch2, Notch3, Notch4 and Cox2 expression in MSCs with or without LPS (100 ng/mL)/IFN-γ (10 ng/mL) stimulation. Representative of three experiments. **D** Western blot analysis of Notch2 and COX2 protein expression in MSCs with or without LPS (100 ng/mL)/IFN-γ (10 ng/mL) stimulation. (E, F) MSCs were transfected with Notch2 siRNA (siNotch2), non-specific siRNA (siNS) or COX2-siRNA (siCOX2) for 48 h. Some cells were cultured on JAG1-coated (10 μg/ml) culture plates for 24 h. **E** Western blot analysis of COX2 expression in MSCs. **F** ELISA analysis of PGE2 levels in the supernatants from MSCs. The data are means ± SD of three independent experiments. **G** Representative histological staining (H&E) of liver sections from MSC-siNS-, MSC-siCOX2-, MSC-siNotch2-, MSC-siNS + JAG1- or MSC-siNotch2 + JAG1-treated animals (*n* = 5 mice/group). Scale bars: 100 μm. **H** Hepatocellular function, as assessed by serum ALT levels (IU/L) (*n* = 5 mice/group). **I** Animal survival curves after a single dose of APAP (650 mg/kg, i.p.) injection over 72 h (*n* = 10 mice/group). All data represent the mean ± SD. *p < 0.05,**p < 0.01

We used a mouse model of APAP-induced acute liver injury to test the therapeutic potential of MSCs in vivo. To track the distribution of MSCs in the injured liver, MSCs were predyed with CellTracker™ CMFDA. As expected, increased number of MSCs (green-fluorescent) were recruited to the injured livers compared with the controls (Additional file 2: Fig. 1). Compared to those in the PBS or fibroblast-treated controls, MSCs-treated livers showed a decreased necrotic area (Fig. 1A), and lower serum ALT levels (IU/L) (Fig. 1B). We then investigated whether the Notch signaling pathway played a key role in the protective effect of MSCs. We analyzed the expressions of all Notch receptors and COX2 genes in LPS/IFNγ-stimulated MSCs. As shown in Fig. 1C, the mRNA levels of Notch2 and COX2, but not Notch1, Notch3 and Notch4, were dramatically upregulated in MSCs after LPS/IFNγ stimulation for 6 h or 12 h. Moreover, Western blot analysis confirmed that the protein levels of Notch2 and COX2 were obviously increased in LPS/IFNγ-stimulated MSCs compared to that in untreated controls (Fig. 1D). To determine whether Notch2 may affect COX2/PGE2 activation in MSCs, we disrupted Notch2 in MSCs with Notch2 siRNA transfection. As expected, Notch2 knockdown in MSCs significantly reduced COX2 expression (Fig. 1E). Moreover, treatment of recombinant Notch ligand JAG1 increased COX2 expression (Fig. 1E). Consistent with the data, JAG1 treatment markedly increased PGE2 secretion in MSCs, whereas Notch2 or COX2 knockdown decreased PGE2 secretion (Fig. 1F).

Next, we tested whether Notch activation enhanced the protective effect of MSCs against APAP-induced liver damage. Indeed, administration of JAG1-pretreated MSCs (JAG1-MSCs) resulted in further improvement of histological damage and hepatocellular function as compared with MSCs-treated controls (Fig. 1G and H). In contrast, administration of COX2-silenced MSCs aggravated APAP-induced liver damage as compared with the MSCs-treated controls (Fig. 1G and H). Importantly, Notch2 knockdown in MSCs markedly diminished the protective effect of MSCs against APAP-induced acute liver injury, even in the presence of JAG1 pretreatment (Fig. 1G and H). Moreover, analysis of survival rate showed a decreased mortality in MSC-treated mice, which was further improved by administration of JAG1-MSCs. However, Notch2 disruption in MSCs diminished this protective effect (Fig. 1I). Taken together, these results demonstrate the key role of Notch2/COX2 axis in MSCs-mediated protective effect against APAP-induced liver injury.

### Notch-activated MSCs reduce macrophage/neutrophil infiltration and hepatocellular apoptosis after APAP challenge

**Fig. 2:**
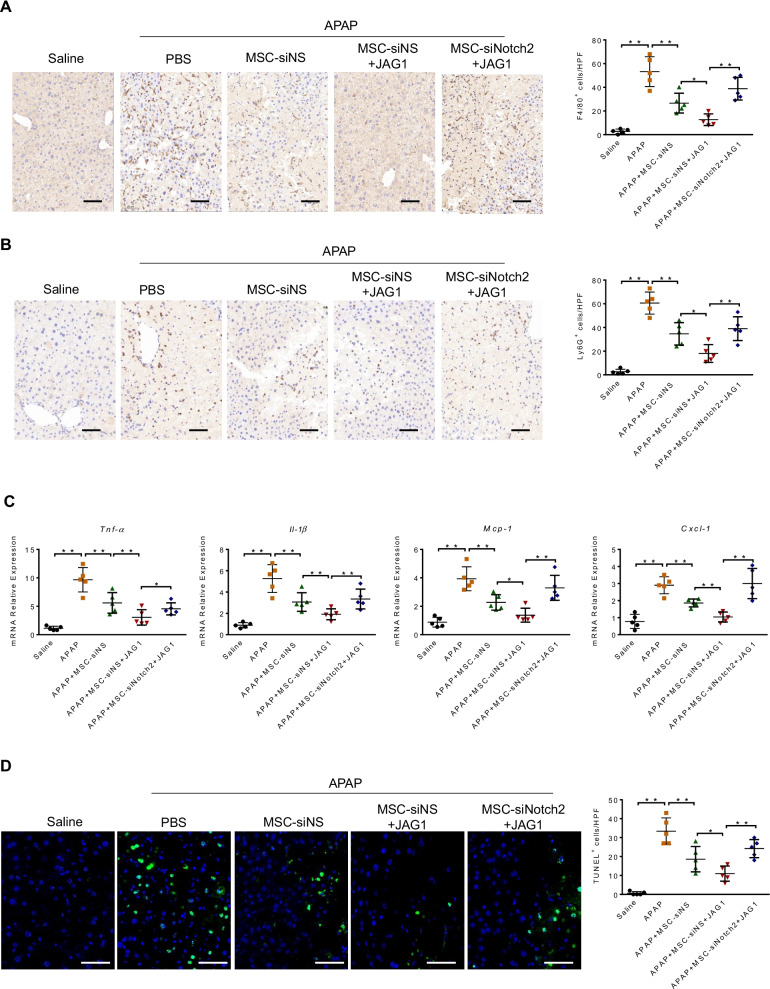
Notch-activated MSCs reduce macrophage/neutrophil infiltration and hepatocellular apoptosis after APAP challenge. **A**, **B** Immunohistochemistry staining and quantification of F4/80^+^ macrophages and Ly6G^+^ neutrophils in livers (*n* = 5 mice/group). Scale bars: 50 μm. **C** Quantitative RT-PCR-assisted detection of TNF-α, Il-1β, Mcp-1 and Cxcl-1 expression. **D** Representative images of TUNEL-staining liver sections and quantification of TUNEL-positive nuclei per high-power field (HPF) for at least 10 fields per sample (*n* = 5 mice/group). Scale bars, 50 μm. Data are presented as the mean ± SD. *p < 0.05, **p < 0.01

To determine whether Notch-activated MSCs affected inflammatory cell infiltration in APAP-challenged livers, F4/80^+^ macrophages and Ly6G^+^ neutrophils were detected by immunohistochemistry. Compared to those in the PBS-treated controls, MSCs-treated livers revealed decreased F4/80^+^ macrophage and Ly6G^+^ neutrophil accumulation, which were further reduced by treatment with JAG1-MSCs (Fig. 2A and B). However, administration of Notch2 siRNA-transfected JAG1-MSCs resulted in increased infiltration of macrophages/neutrophils as compared with NS siRNA-transfected controls (Fig. 2A and B). Consistent with these data, MSC treatment reduced the mRNA levels of proinflammatory cytokines and chemokines including TNF-α, IL-1β, MCP-1 and CXCL-1 in liver tissues compared to that in PBS-treated controls (Fig. 2C). Similarly, JAG1-MSC treatment further decreased inflammatory mediators, whereas Notch2 silence abolished the beneficial effect of JAG1-MSCs on APAP-induced liver inflammation (Fig. 2C).

Next, we investigated the effects of MSCs and JAG1-MSCs on hepatocellular apoptosis associated with APAP-induced liver damage. TUNEL staining showed that MSC administration decreased the frequency of TUNEL^+^ cells in injured livers, and JAG1 stimulation enhanced the ability of MSCs to suppress hepatocellular apoptosis, which was eliminated by Notch2 knockdown in MSCs (Fig. 2D).

### Notch-activated MSCs inhibit XBP1s/NLRP3 activation in APAP-induced liver injury

**Fig. 3:**
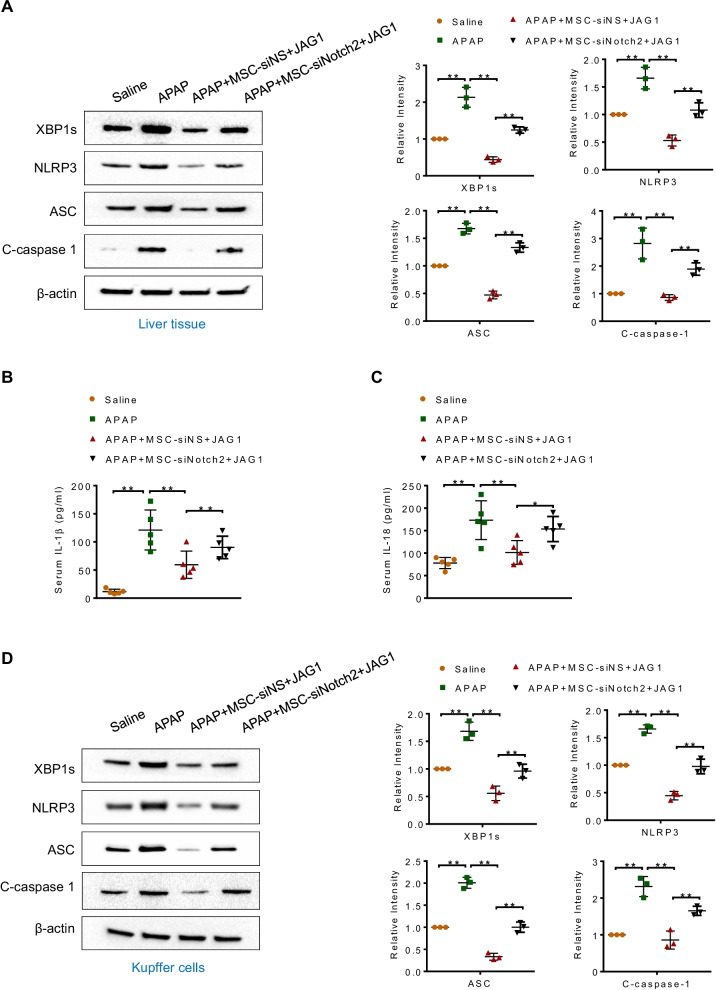
Notch-activated MSCs inhibit XBP1s/NLRP3 activation in APAP-induced liver injury. **A** Western blot analysis of XBP1s, NLRP3, ASC and C-caspase-1 expression in liver tissues from the indicated animals. **B**, **C** ELISA analysis of serum IL-1β and IL-18 levels (*n* = 5 mice/group). **D** Hepatic macrophages (Kupffer cells) were isolated from mice after APAP injection, with or without MSC-siNS + JAG1 or MSC-siNotch2 + JAG1 treatment. Western blot analysis of XBP1s, NLRP3, ASC and C-caspase-1 expression in Kupffer cells. Representative of three experiments. Data are presented as the mean ± SD. *p < 0.05, **p < 0.01

Previous studies have demonstrated that XBP1s is essential for the activation of NLRP3 inflammasome in response to inflammatory stimuli [25, 26]. Our recent study indicated that murine bone marrow-derived MSCs attenuated ischemia/reperfusion-induced liver inflammatory injury through controlling NLRP3 activation [9]. Thus, we speculated that Notch-activated MSCs may influence XBP1s/NLRP3-driven inflammatory response during APAP-induced acute liver injury. As expected, administration of JAG1-MSCs significantly inhibited XBP1s, NLRP3, ASC, cleaved caspase-1 expression (Fig. 3A) and reduced serum IL-1β (Fig. 3B) and IL-18 (Fig. 3C) levels, which were abolished by Notch2 disruption in MSCs (Fig. 3A–C). To determine whether MSCs specifically influence XBP1s/NLRP3 activation in hepatic macrophages, we isolated Kupffer cells from APAP-injected livers with or without MSC treatment (Additional file 3: Fig. 2). Indeed, MSC treatment reduced XBP1s, NLRP3, ASC and cleaved caspase-1 expression in hepatic macrophages (Fig. 3D). However, these decreased expressions were markedly reversed when Notch2 was blocked in JAG1-MSCs (Fig. 3D).

### XBP1s repression is crucial for the anti-inflammatory effect of MSCs in APAP-induced liver injury

**Fig. 4:**
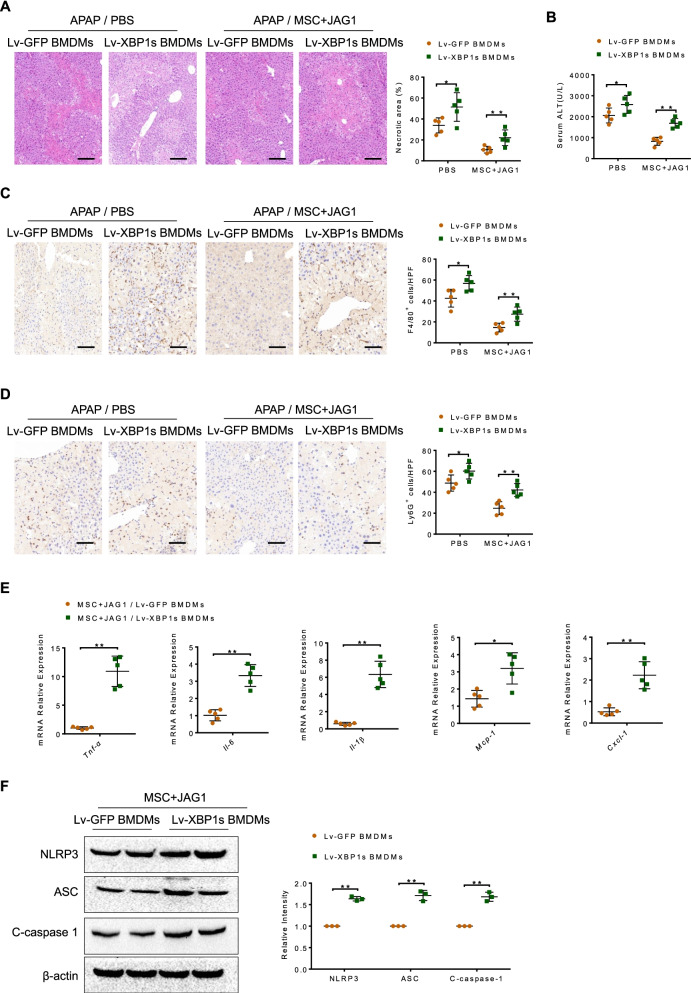
XBP1s repression is crucial for the anti-inflammatory effect of MSCs in APAP-induced liver injury. Bone marrow-derived macrophages (BMDMs; 5 × 10^6^ cells/mouse) were transfected with lentivirus expressing XBP1s (Lv-XBP1s) or GFP control (Lv-GFP) and adoptively transferred into JAG1-MSC-treated mice 24 h prior to APAP injection. **A** Representative histological staining (H&E) of liver tissue (*n* = 5 mice/group) and percent of necrotic area. Scale bars: 100 μm. **B** Hepatocellular function, as assessed by serum ALT levels (IU/L) (*n* = 5 mice/group). **C**, **D** Immunohistochemistry staining and quantification of F4/80^+^ macrophages and Ly6G^+^ neutrophils in livers (*n* = 5 mice/group). Scale bars: 50 μm. **E** Quantitative RT-PCR-assisted detection of TNF-α, Il-6, Il-1β, Mcp-1 and Cxcl-1 expression. **F** Western blot analysis of NLRP3, ASC and C-caspase-1 expression in liver tissues. Data are presented as the mean ± SD. *p < 0.05, **p < 0.01

We then tested whether XBP1s were required for MSCs-mediated anti-inflammatory effect in APAP-induced liver injury. BMDMs were transfected with lentivirus expressing XBP1s (Lv-XBP1s) or GFP control (Lv-GFP) and adoptively transferred into JAG1-MSC-treated mice (Additional file 4: Fig. 3). Clearly, adoptive transfer of Lv-XBP1s-transfected BMDMs into JAG1-MSC-treated mice aggravated APAP-induced liver damage, as manifested by increased necrotic area (Fig. 4A), serum ALT level (Fig. 4B), F4/80^+^ macrophage (Fig. 4C) and Ly6G^+^ neutrophil (Fig. 4D) infiltration and pro-inflammatory mediator expression (Fig. 4E). Moreover, unlike livers treated with Lv-GFP-transfected control cells, livers in JAG1-MSC-injected mice treated with Lv-XBP1s-transfected BMDMs revealed augmented NLRP3, ASC and cleaved caspase-1 expression (Fig. 4F). These results suggest that administration of Notch-activated MSCs alleviates APAP-induced hepatotoxicity and inflammatory response through inhibiting XBP1/NLRP3 inflammasome activation.

### Notch-activated MSCs inhibit XBP1s/NLRP3 activation in an AMPK/SIRT1-dependent manner

**Fig. 5:**
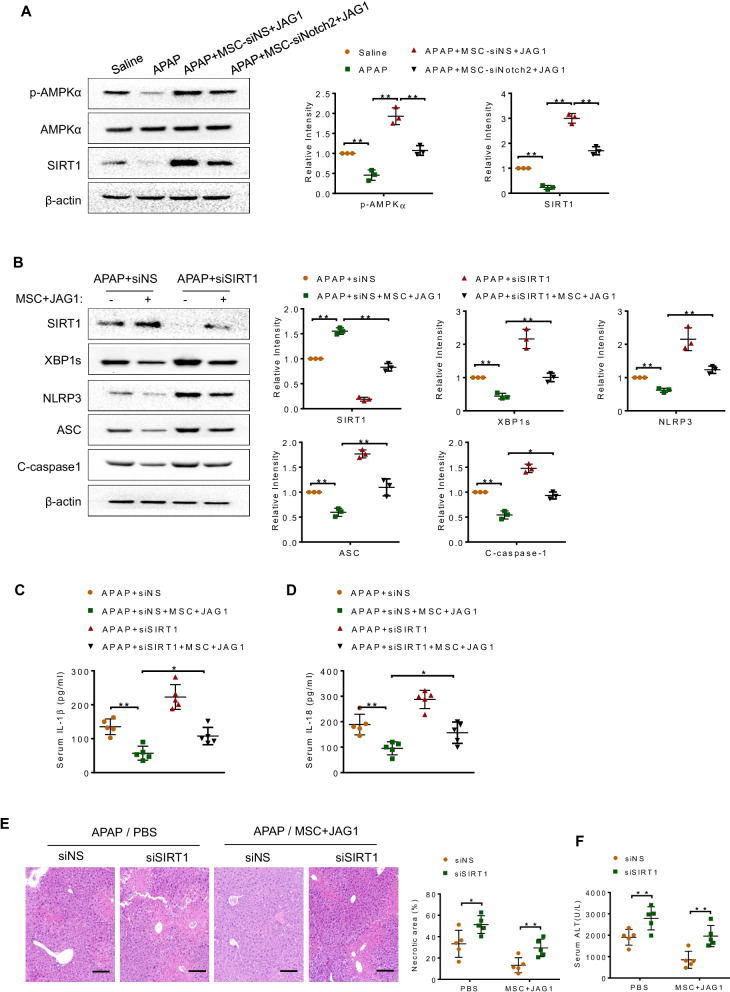
Notch-activated MSCs inhibit XBP1s/NLRP3 activation in an AMPK/SIRT1-dependent manner. JAG1-MSCs-treated mice were injected via the tail vein with SIRT1 siRNA (siSIRT1; 2 mg/kg) or non-specific control siRNAs (siNS) mixed with mannose-conjugated polymers 4 h prior to drug induction. **A** Western blot analysis of p-AMPKα, AMPKα and SIRT1 expression in liver tissues. **B** Western blot analysis of SIRT1, XBP1s, NLRP3, ASC and C-caspase-1 protein expression in liver tissues. **C**, **D** ELISA analysis of serum IL-1β and IL-18 levels. **E** Representative histological staining (H&E) of liver tissue (*n* = 5 mice/group) and percent of necrotic area. Scale bars: 100 μm. **F** Hepatocellular function, as assessed by serum ALT levels (IU/L). Data are presented as the mean ± SD. *p < 0.05, **p < 0.01

As SIRT1 has been identified to deacetylate XBP1s and inhibit its transcriptional activity [27, 28], we then asked whether MSCs may regulate XBP1s/NLRP3 activation through SIRT1-dependent pathway. We first analyzed whether MSCs influenced AMPK/SIRT1 signaling in APAP-injured livers. APAP challenge resulted in downregulation of AMPKα phosphorylation and SIRT1 expression (Fig. 5A). Administration of JAG1-MSCs markedly enhanced p-AMPKα and SIRT1 expression, whereas Notch2 disruption in JAG1-MSCs diminished p-AMPKα and SIRT1 expression (Fig. 5A). Next, we disrupted SIRT1 in JAG1-MSC-treated livers using SIRT1 siRNA with an in vivo mannose-mediated delivery system that enhances delivery to cells expressing a mannose-specific membrane receptor to macrophages as previously described by ours and others [23, 29, 30]. Unlike NS siRNA-treated controls, SIRT1 siRNA treatment augmented XBP1s, NLRP3 and cleaved caspase-1 expression in the JAG1-MSC-treated livers (Fig. 5B), accompanied by increased serum IL-1β and IL-18 levels (Fig. 5C and D). Moreover, SIRT1 knockdown in the JAG1-MSC-treated mice aggravated APAP-induced hepatotoxicity, evidenced by increased necrotic area (Fig. 5E) and serum ALT levels (Fig. 5F), compared to the NS siRNA-treated controls. These results suggest that AMPK/SIRT1 axis is required for MSCs-mediated regulation of XBP1s/NLRP3 function in APAP-induced liver inflammatory response.

### Notch-activated MSCs promote XBP1s deacetylation and inhibit XBP1s/NLRP3 activation in macrophages

**Fig. 6:**
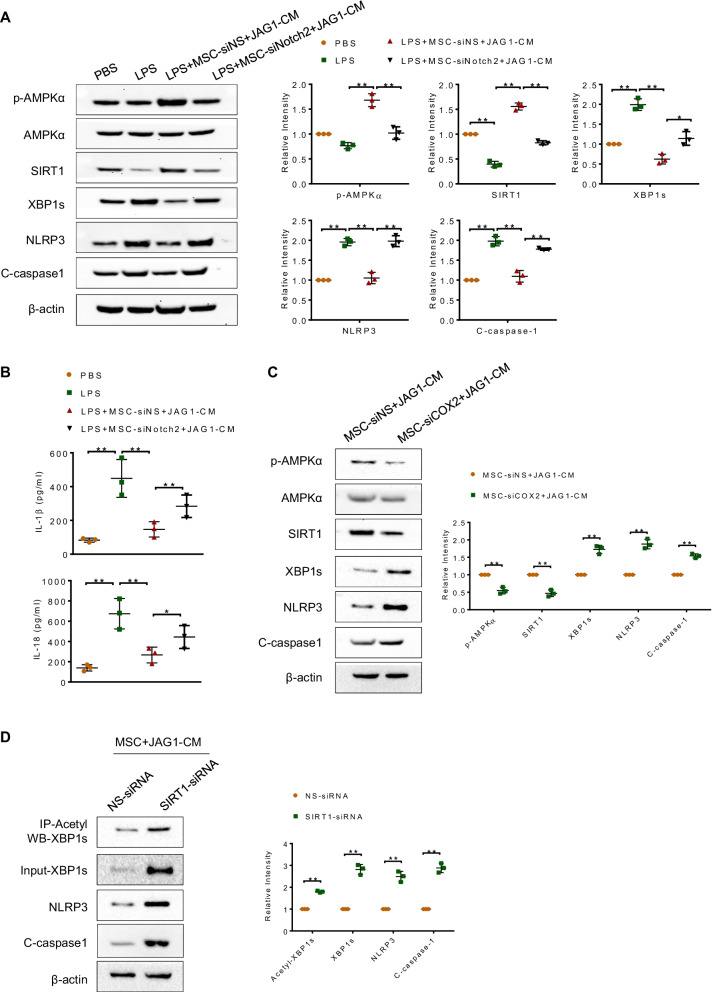
Notch-activated MSCs promote XBP1s deacetylation and inhibit XBP1s/NLRP3 activation in macrophages. Bone marrow-derived macrophages (BMDMs, 1 × 10^6^) were treated with MSC-derived conditioned media (MSC-CM) for 24 h followed by LPS (100 ng/ml) stimulation. **A** Western blot analysis of p-AMPKα, AMPKα, SIRT1, XBP1s, NLRP3 and C-caspase-1 expression in BMDMs. **B** ELISA analysis of IL-1β and IL-18 levels from BMDM supernatant. **C** Western blot analysis of p-AMPKα, AMPKα, SIRT1, XBP1s, NLRP3 and C-caspase-1 expression in BMDMs. **D** BMDMs were transfected with NS siRNA or SIRT1 siRNA and then treated with JAG1-MSC-CM. The XBP1s acetylation level was evaluated by performing immunoprecipitation (IP) with anti-acetylation antibody and Western blotting with anti-XBP1s antibody. The expressions of XBP1s, NLRP3 and C-caspase-1 in the whole-cell lysates were examined by Western blot analysis. All data are presented as means ± SD. *p < 0.05; **p < 0.01

To elucidate the mechanistic role of the Notch2/COX2/SIRT1 pathway in the control of XBP1/NLRP3 inflammasome activation in MSCs-mediated immunomodulation, bone marrow-derived macrophages (BMDMs) were cultured with conditioned media (CM) from JAG1-MSCs transfected with Notch2 siRNA or COX2-siRNA. Notably, Notch activation in MSCs by JAG1 promoted AMPKα phosphorylation, SIRT1 expression and inhibited XBP1s, NLRP3, cleaved caspase-1 expression in BMDMs after LPS stimulation. However, Notch2 knockdown in JAG1-MSCs depressed AMPKα phosphorylation, SIRT1 expression while enhancing XBP1s, NLRP3 and cleaved caspase-1 expression in macrophages (Fig. 6A). Consistently, JAG1-MSC treatment reduced IL-1β and IL-18 production in macrophages, which was abolished by Notch2-silenced JAG1-MSCs (Fig. 6B). In addition, we analyzed whether COX2 was responsible for MSCs-mediated AMPK/SIRT1 activation and subsequent XBP1s/NLRP3 inhibition. Indeed, COX2 knockdown in JAG1-MSCs significantly inhibited AMPKα phosphorylation, SIRT1 expression, but augmented XBP1s, NLRP3 and cleaved caspase-1 expression in macrophages (Fig. 6C). Furthermore, we examined whether SIRT1 was essential for MSCs-mediated XBP1s deacetylation in macrophages. As expected, unlike NS siRNA-treated controls, SIRT1 disruption reduced deacetylation of XBP1s and promoted XBP1s, NLRP3 and cleaved caspase-1 expression in JAG1-MSC-treated macrophages (Fig. 6D). Collectively, these results suggest that Notch2/COX2 axis in MSCs induces AMPK/SIRT1 activation, leading to XBP1s deacetylation and NLRP3 inflammasome inhibition in macrophages.

## Discussion

It is well documented that many types of innate and adaptive immune cells are present at sites of inflammation and can be regulated by MSCs [6, 7]. In particular, hepatic macrophages are essential pathogenic drivers for liver inflammation, fibrosis and hepatocarcinogenesis, and thus, they are an attractive target for novel therapeutic approaches to treat liver diseases [5]. The present study demonstrates that MSCs-specific Notch signaling regulates macrophage XBP1s/NLRP3 activation for orchestrating inflammatory responses in APAP-induced acute liver injury. First, MSC administration significantly alleviated APAP-induced liver injury. Moreover, the anti-inflammatory activity and therapeutic effects of MSCs were greatly enhanced by Notch activation. However, Notch2 or COX2 disruption in MSCs diminished the protective effect of MSCs against liver damage. These results demonstrate that Notch2/COX2 axis is crucial for the ability of MSCs to reduce the severity of APAP-induced liver injury in mice. Second, Notch-activated MSCs induced macrophage AMPK/SIRT1 activation, which in turn inhibited XBP1s/NLRP3 inflammasome activity. Furthermore, SIRT1/XBP1s signaling was required for MSCs-mediated immunosuppression and protective effect against APAP-induced liver damage. Third, mechanistic studies further demonstrated that MSCs-mediated AMPK/SIRT1 activation in macrophages resulted in XBP1s deacetylation and subsequent inhibition of NLRP3 inflammasome. These findings highlight the importance of Notch2/COX2/AMPK/SIRT1 pathway in MSCs-mediated immunomodulation during liver inflammation.

The discovery that MSCs contribute to tissue repair due to their immunomodulatory and regenerative potentials has revolutionized stem cell therapy for the treatment of inflammatory diseases [31]. Although much progress has been made in the use of MSCs to treat various diseases, many of them have shown therapeutic failure, especially in humans [32]. Thus, exploring the underlying immunomodulatory mechanisms that govern the immunosuppressive potential of MSCs will lead to the development of novel therapeutic strategies for inflammatory diseases. Accumulating evidence demonstrates that Notch signaling can regulate cell fate decisions including proliferation, lineage commitment and terminal differentiation in various adult stem cells [10, 13–17]. A previous report indicated that blocking Notch signaling in MSCs with two inhibitors γ-secretase decreased COX2 expression and PGE2 secretion [22]. The main finding of this study was that JAG1-mediated Notch signaling promoted COX2/PGE2 production and enhanced the protective effects of MSCs against APAP-induced acute liver injury. Furthermore, our study showed that Notch2 disruption in JAG1-pretreated MSCs significantly reversed the protective effect of MSCs, suggesting that Notch2 activation was required for the immunomodulatory capabilities of MSCs. In addition, Notch-activated MSCs reduced macrophage/neutrophil infiltration in the injured liver, at least in part, through inhibiting the expression of chemokines including MCP-1 and CXCL-1. These findings support the hypothesis that JAG1-activated Notch signaling pathway in MSCs may serve as a novel strategy to enhance their therapeutic potentials in vivo owing to the elevated production of PGE2. Indeed, multiple lines of evidence from our own and other groups demonstrates that PGE2 is one of the most potent therapeutic soluble factors of MSCs and mediates most of the immunomodulatory capacity [7, 9, 22, 33, 34]. Although the present study clearly showed the importance of Notch2 in COX2 expression and PGE2 secretion by MSCs, the exact mechanism by which Notch2 regulates COX2 expression in MSCs remains to be determined.

Another important implication of the present study is that inhibition of XBP1/NLRP3 inflammasome contributes to MSCs-mediated immunomodulation in APAP-induced liver inflammation. It is well known that the NLRP3 inflammasome is a complex which is composed of a series of protein such as the NOD-like receptor NLRP3, the adaptor molecule apoptosis-associated speck-like protein containing a caspase recruitment domain (ASC) and the enzyme caspase-1 [35–38]. The biochemical function of NLRP3 inflammasomes is to activate caspase-1, which leads to the maturation of IL-1β and IL-18 [39]. The importance of the NLRP3 inflammasome in immunity and various inflammatory diseases including acute or chronic liver inflammation has been well documented [9, 25, 37, 40]. Several regulators of NLRP3 inflammasome activation, including double-stranded RNA-dependent protein kinase, guanylate-binding protein 5 and NEK7, have been reported [36]. However, a unified mechanism has not yet emerged. Recently, data from other groups as well as our own showed that XBP1s promoted NLRP3 inflammasome activation in acute and chronic liver inflammation [9, 25, 26, 41]. XBP1s has multiple roles in regulating the immune response. Previous studies have demonstrated that Toll-like receptors specifically activated the endoplasmic reticulum stress sensor kinase IRE1ɑ and its downstream target, the transcription factor XBP1s, which identified a critical function for XBP1s in innate immune responses in macrophages [42]. In line with these findings, our present data showed that adoptive transfer of Lv-XBP1s-transfected macrophages into MSC-treated mice augmented NLRP3 inflammasome activation and exacerbated APAP-induced liver damage. These findings proved that XBP1s repression is crucial for the anti-inflammatory effect of MSCs in APAP-induced liver damage. The present study further supports the notion that the development of pharmacological inhibitors of the XBP1/NLRP3 pathway could present a new therapeutic strategy aimed at diminishing inflammatory liver diseases.

Given the demonstrated role of XBP1/NLRP3 inflammasome in MSCs-mediated immunomodulation, we then investigated how MSCs could influence XBP1/NLRP3 activation in APAP-induced acute liver injury. Several previous studies showed that PGE2 was observed to significantly induce the phosphorylation of AMPKα (Thr-172) in various cell types, including bone marrow-derived cells [43–45]. It is currently recognized that the phosphorylation at site Thr-172 of the α-subunit is essential for AMPK activation [46], and that AMPK is a central regulator of multiple metabolic pathways and may have therapeutic importance for treating metabolic diseases including obesity, type 2 diabetes, non-alcoholic fatty liver disease and cardiovascular disease [47]. Importantly, the deacetylase SIRT1, a target of p-AMPK, has marked anti-inflammatory effects in diverse tissues and cell models [48–51]. Additionally, it has been reported that macrophage AMPK activation promoted the deacetylase SIRT1 expression [52], and that SIRT1 inhibited inflammatory pathways in macrophages and modulated insulin sensitivity [53]. Based on these findings, we speculated that MSC-secreted PGE2 could activate AMPK/SIRT1 pathway in macrophages, leading to attenuation of liver inflammation. As expected, our results indicated that APAP challenge resulted in downregulation of p-AMPK and SIRT1; however, administration of JAG1-MSCs markedly enhanced AMPK/SIRT1 activation in macrophages. Furthermore, SIRT1 knockdown in the JAG1-MSC-treated mice augmented XBP1/NLRP3 activation and exacerbated liver damage. These results suggest that AMPK/SIRT1 activation is required for MSCs-mediated inhibition of XBP1/NLRP3 and attenuation of liver damage. Moreover, our data showed that Notch2 disruption in JAG1-MSCs diminished AMPK/SIRT1 activation and enhanced XBP1/NLRP3 activity in macrophages, suggesting an important role of Notch2 in MSCs-mediated AMPK/SIRT1 activation.

SIRT1, as a nicotinamide adenine dinucleotide-dependent deacetylase, can deacetylate histones, non-histones and other transcription factors, which participates in various physiological functions including cell aging, gene transcription, energy balance and regulation of oxidative stress [54, 55]. Recent evidence demonstrates that SIRT1 is a key player in liver physiology and a therapeutic target against liver inflammation including hepatic ischemia/reperfusion injury and APAP-induced liver injury [56, 57]. Here, we demonstrated that MSCs promoted AMPK/SIRT1 activation, leading to XBP1s deacetylation and NLRP3 inflammasome inhibition in macrophages. This result is consistent with a recent report that SIRT1 ameliorated cadmium-induced endoplasmic reticulum stress and NLRP3 inflammasome-dependent pyroptosis through XBP1s deacetylation in human renal tubular epithelial cells [28]. Taken together, these findings suggest that AMPK/SIRT1 activation may be of therapeutic benefit for the treatment of inflammatory diseases.

## Conclusion

**Fig. 7:**
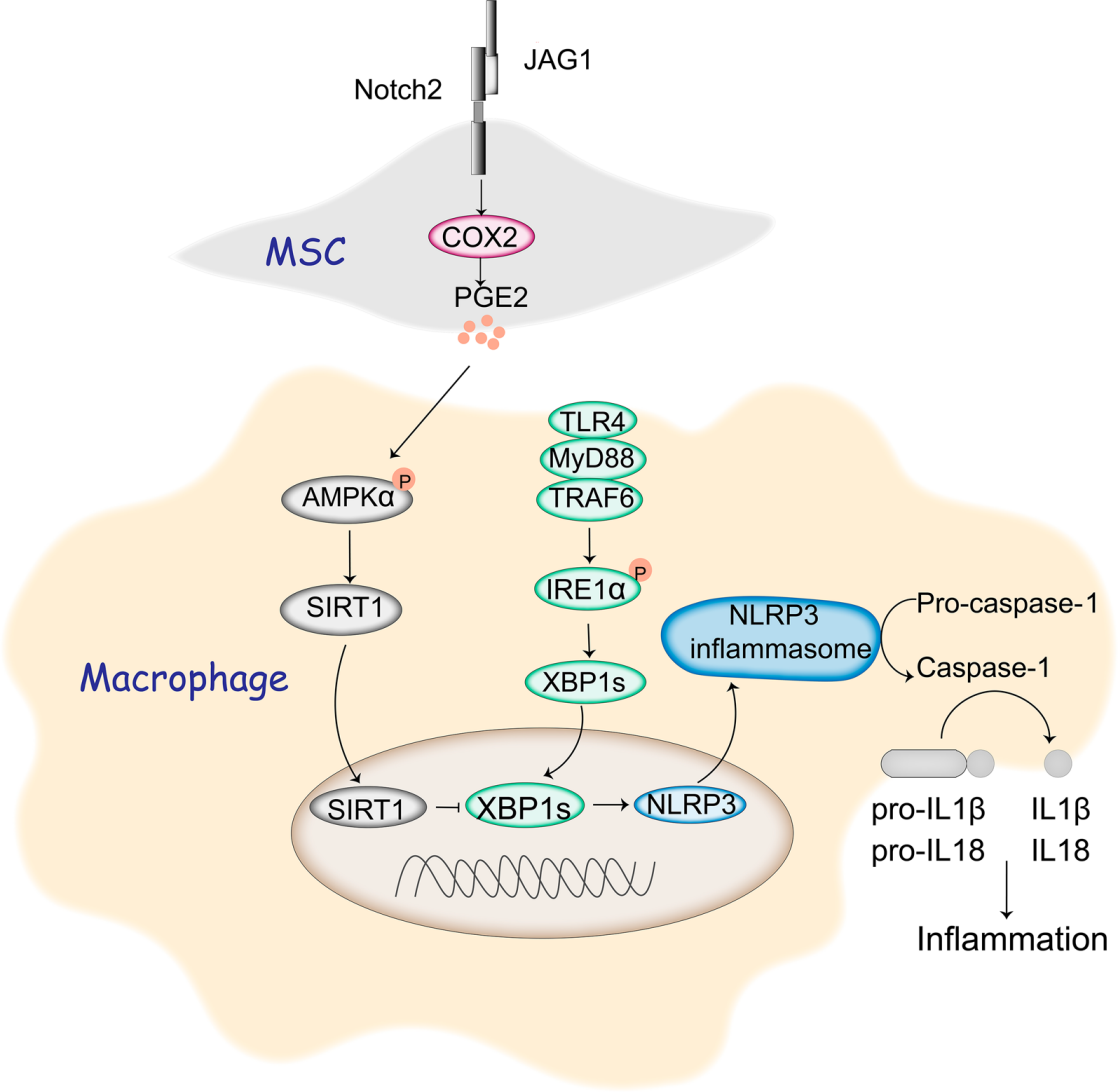
Schematic illustration of MSCs-mediated immunomodulation by activating Notch2/COX2/AMPK/SIRT1 pathway. Notch activation in MSCs by its ligand Jagged1 (JAG1) increased cyclooxygenase-2 (COX2) expression and prostaglandin E2 (PGE2) secretion, enhanced AMP-activated protein kinase (AMPKα) phosphorylation and the sirtuins 1 (SIRT1) deacetylase expression in macrophages, resulting in deacetylation of spliced X-box-binding protein 1 (XBP1s) and subsequent inhibition of NLR family pyrin domain-containing 3 (NLRP3) inflammasome during liver inflammation

We identify a key role of Notch2/COX2/AMPK/SIRT1 pathway in controlling XBP1/NLRP3 activation in MSCs-mediated immunomodulation. Our data indicate that Notch activation enhances the protective effect of MSCs against APAP-induced liver damage. We further demonstrate that Notch-activated MSCs promote COX2/PGE2 production, which in turn activate AMPK/SIRT1 pathway, leading to XBP1s deacetylation and subsequent inhibition of NLRP3 inflammasome in macrophages (Fig. 7). Thus, our findings suggest an effective strategy to prevent and/or treat liver inflammatory diseases by modulating the AMPK/SIRT1 pathway and targeting the XBP1/NLRP3 activity in macrophages using genetically modified MSC approaches or pharmacological interventions.

## Supplementary Information

**Additional file 1: Table S1.** Primer sequences for the amplification.

**Additional file 2: Fig. S1.** Tracking of MSCs in the liver. Mice were injected through the tail vein with CellTrackerTM green CMFDA predyed MSCs (1X10^6^) 24 h prior to APAP (400 mg/kg, i.p.) or saline injection. Representative immunofluorescence images for the MSCs labeled with CMFDA (green) localized in the liver. DAPI was used to visualize nuclei (blue). Scale bars: 20 μm.

**Additional file 3: Fig. S2.** Identification of isolated Kupffer cells. Hepatic macrophages (Kupffer cells) were isolated from mice. Representative immunofluorescence staining for F4/80 (red). DAPI was used to visualize nuclei (blue). Scale bars: 40 μm.

**Additional file 4: Fig. S3.** Immunofluorescence staining of GFP-labeled and F4/80^+^ macrophages in liver tissue. Bone marrow-derived macrophages (BMDMs; 5×10^6^ cells/mouse) were transfected with lentivirus expressing GFP (Lv-GFP) and adoptively transferred into mice 24 h prior to APAP injection. DAPI was used to visualize nuclei (blue). Scale bars: 100 μm.

## Data Availability

The data that support the findings of this study can be obtained from the corresponding author upon reasonable request.

## Abbreviations

BMDMs: Bone marrow-derived macrophages
COX2: Cyclooxygenase-2
LPS: Lipopolysaccharide
MSCs: Mesenchymal stromal/stem cells
PGE2: Prostaglandin E2
siRNA: Small interfering RNA
ALT: Alanine aminotransferase
NLRP3: NOD-like receptor family pyrin domain-containing 3
SIRT1: Sirtuins 1
AMPK: AMP-activated protein kinase
XBP1s: Spliced X-box-binding protein 1

## References

[1] Larson AM, Polson J, Fontana RJ, Davern TJ, Lalani E, Hynan LS (2005). Acetaminophen-induced acute liver failure: results of a United States multicenter, prospective study. Hepatology.

[2] Fisher JE, McKenzie TJ, Lillegard JB, Yu Y, Juskewitch JE, Nedredal GI (2013). Role of kupffer cells and toll-like receptor 4 in acetaminophen-induced acute liver failure. J Surg Res.

[3] Antoniades CG, Quaglia A, Taams LS, Mitry RR, Hussain M, Abeles R (2012). Source and characterization of hepatic macrophages in acetaminophen-induced acute liver failure in humans. Hepatology.

[4] Biagioli M, Carino A, Fiorucci C, Marchiano S, Di Giorgio C, Bordoni M (2020). The bile acid receptor GPBAR1 modulates CCL2/CCR2 signaling at the liver sinusoidal/macrophage interface and reverses acetaminophen-induced liver toxicity. J Immunol.

[5] Tacke F (2017). Targeting hepatic macrophages to treat liver diseases. J Hepatol.

[6] Wang LT, Ting CH, Yen ML, Liu KJ, Sytwu HK, Wu KK (2016). Human mesenchymal stem cells (MSCs) for treatment towards immune- and inflammation-mediated diseases: review of current clinical trials. J Biomed Sci.

[7] Shi Y, Wang Y, Li Q, Liu K, Hou J, Shao C (2018). Immunoregulatory mechanisms of mesenchymal stem and stromal cells in inflammatory diseases. Nat Rev Nephrol.

[8] Owen A, Newsome PN (2015). Mesenchymal stromal cell therapy in liver disease: opportunities and lessons to be learnt?. Am J Physiol Gastrointest Liver Physiol.

[9] Li C, Jin Y, Wei S, Sun Y, Jiang L, Zhu Q (2019). Hippo signaling controls NLR family pyrin domain containing 3 activation and governs immunoregulation of mesenchymal stem cells in mouse liver injury. Hepatology.

[10] Dong Y, Long T, Wang C, Mirando AJ, Chen J, O'Keefe RJ (2014). NOTCH-mediated maintenance and expansion of human bone marrow stromal/stem cells: a technology designed for orthopedic regenerative medicine. Stem Cells Transl Med.

[11] Radtke F, Fasnacht N, Macdonald HR (2010). Notch signaling in the immune system. Immunity.

[12] Kopan R, Ilagan MX (2009). The canonical Notch signaling pathway: unfolding the activation mechanism. Cell.

[13] Imayoshi I, Sakamoto M, Yamaguchi M, Mori K, Kageyama R (2010). Essential roles of Notch signaling in maintenance of neural stem cells in developing and adult brains. J Neurosci.

[14] Jensen J, Pedersen EE, Galante P, Hald J, Heller RS, Ishibashi M (2000). Control of endodermal endocrine development by Hes-1. Nat Genet.

[15] Mourikis P, Sambasivan R, Castel D, Rocheteau P, Bizzarro V, Tajbakhsh S (2012). A critical requirement for notch signaling in maintenance of the quiescent skeletal muscle stem cell state. Stem Cells.

[16] Stier S, Cheng T, Dombkowski D, Carlesso N, Scadden DT (2002). Notch1 activation increases hematopoietic stem cell self-renewal in vivo and favors lymphoid over myeloid lineage outcome. Blood.

[17] Varnum-Finney B, Xu L, Brashem-Stein C, Nourigat C, Flowers D, Bakkour S (2000). Pluripotent, cytokine-dependent, hematopoietic stem cells are immortalized by constitutive Notch1 signaling. Nat Med.

[18] Radtke F, MacDonald HR, Tacchini-Cottier F (2013). Regulation of innate and adaptive immunity by Notch. Nat Rev Immunol.

[19] Lu L, Yue S, Jiang L, Li C, Zhu Q, Ke M (2018). Myeloid Notch1 deficiency activates the RhoA/ROCK pathway and aggravates hepatocellular damage in mouse ischemic livers. Hepatology.

[20] Yu HC, Qin HY, He F, Wang L, Fu W, Liu D (2011). Canonical notch pathway protects hepatocytes from ischemia/reperfusion injury in mice by repressing reactive oxygen species production through JAK2/STAT3 signaling. Hepatology.

[21] Jin Y, Li C, Xu D, Zhu J, Wei S, Zhong A (2020). Jagged1-mediated myeloid Notch1 signaling activates HSF1/Snail and controls NLRP3 inflammasome activation in liver inflammatory injury. Cell Mol Immunol.

[22] Bartosh TJ, Ylostalo JH, Bazhanov N, Kuhlman J, Prockop DJ (2013). Dynamic compaction of human mesenchymal stem/precursor cells into spheres self-activates caspase-dependent IL1 signaling to enhance secretion of modulators of inflammation and immunity (PGE2, TSG6, and STC1). Stem Cells.

[23] Zhu Q, Wang H, Jiang B, Ni X, Jiang L, Li C (2018). Loss of ATF3 exacerbates liver damage through the activation of mTOR/p70S6K/ HIF-1alpha signaling pathway in liver inflammatory injury. Cell Death Dis.

[24] Xiang E, Han B, Zhang Q, Rao W, Wang Z, Chang C (2020). Human umbilical cord-derived mesenchymal stem cells prevent the progression of early diabetic nephropathy through inhibiting inflammation and fibrosis. Stem Cell Res Ther.

[25] Yue S, Zhu J, Zhang M, Li C, Zhou X, Zhou M (2016). The myeloid heat shock transcription factor 1/beta-catenin axis regulates NLR family, pyrin domain-containing 3 inflammasome activation in mouse liver ischemia/reperfusion injury. Hepatology.

[26] Robblee MM, Kim CC, Porter Abate J, Valdearcos M, Sandlund KL, Shenoy MK (2016). Saturated fatty acids engage an IRE1alpha-dependent pathway to activate the NLRP3 inflammasome in myeloid cells. Cell Rep.

[27] Wang FM, Chen YJ, Ouyang HJ (2011). Regulation of unfolded protein response modulator XBP1s by acetylation and deacetylation. Biochem J.

[28] Chou X, Ding F, Zhang X, Ding X, Gao H, Wu Q (2019). Sirtuin-1 ameliorates cadmium-induced endoplasmic reticulum stress and pyroptosis through XBP-1s deacetylation in human renal tubular epithelial cells. Arch Toxicol.

[29] Li C, Sheng M, Lin Y, Xu D, Tian Y, Zhan Y (2021). Functional crosstalk between myeloid Foxo1-beta-catenin axis and Hedgehog/Gli1 signaling in oxidative stress response. Cell Death Differ.

[30] Yu SS, Lau CM, Barham WJ, Onishko HM, Nelson CE, Li H (2013). Macrophage-specific RNA interference targeting via "click", mannosylated polymeric micelles. Mol Pharm.

[31] Wang Y, Chen X, Cao W, Shi Y (2014). Plasticity of mesenchymal stem cells in immunomodulation: pathological and therapeutic implications. Nat Immunol.

[32] Trounson A, McDonald C (2015). Stem cell therapies in clinical trials: progress and challenges. Cell Stem Cell.

[33] Nemeth K, Leelahavanichkul A, Yuen PS, Mayer B, Parmelee A, Doi K (2009). Bone marrow stromal cells attenuate sepsis via prostaglandin E(2)-dependent reprogramming of host macrophages to increase their interleukin-10 production. Nat Med.

[34] Kim HS, Shin TH, Lee BC, Yu KR, Seo Y, Lee S (2013). Human umbilical cord blood mesenchymal stem cells reduce colitis in mice by activating NOD2 signaling to COX2. Gastroenterology.

[35] Guo C, Xie S, Chi Z, Zhang J, Liu Y, Zhang L (2016). Bile acids control inflammation and metabolic disorder through inhibition of NLRP3 inflammasome. Immunity.

[36] He Y, Hara H, Nunez G (2016). Mechanism and regulation of NLRP3 inflammasome activation. Trends Biochem Sci.

[37] Wree A, Eguchi A, McGeough MD, Pena CA, Johnson CD, Canbay A (2014). NLRP3 inflammasome activation results in hepatocyte pyroptosis, liver inflammation, and fibrosis in mice. Hepatology.

[38] Zhu H, Cao X (2017). NLR members in inflammation-associated carcinogenesis. Cell Mol Immunol.

[39] Mangan MSJ, Olhava EJ, Roush WR, Seidel HM, Glick GD, Latz E (2018). Targeting the NLRP3 inflammasome in inflammatory diseases. Nat Rev Drug Discov.

[40] Mridha AR, Wree A, Robertson AAB, Yeh MM, Johnson CD, Van Rooyen DM (2017). NLRP3 inflammasome blockade reduces liver inflammation and fibrosis in experimental NASH in mice. J Hepatol.

[41] Lebeaupin C, Vallee D, Rousseau D, Patouraux S, Bonnafous S, Adam G (2018). Bax inhibitor-1 protects from nonalcoholic steatohepatitis by limiting inositol-requiring enzyme 1 alpha signaling in mice. Hepatology.

[42] Martinon F, Chen X, Lee AH, Glimcher LH (2010). TLR activation of the transcription factor XBP1 regulates innate immune responses in macrophages. Nat Immunol.

[43] Rowart P, Erpicum P, Krzesinski JM, Sebbagh M, Jouret F (2017). Mesenchymal stromal cells accelerate epithelial tight junction assembly via the AMP-activated protein kinase pathway, independently of liver kinase B1. Stem Cells Int.

[44] Zhu Z, Fu C, Li X, Song Y, Li C, Zou M (2011). Prostaglandin E2 promotes endothelial differentiation from bone marrow-derived cells through AMPK activation. PLoS ONE.

[45] Kainuma S, Otsuka T, Kuroyanagi G, Yamamoto N, Matsushima-Nishiwaki R, Kozawa O (2016). Regulation by AMP-activated protein kinase of PGE2-induced osteoprotegerin synthesis in osteoblasts. Mol Med Rep.

[46] Hawley SA, Davison M, Woods A, Davies SP, Beri RK, Carling D (1996). Characterization of the AMP-activated protein kinase kinase from rat liver and identification of threonine 172 as the major site at which it phosphorylates AMP-activated protein kinase. J Biol Chem.

[47] Day EA, Ford RJ, Steinberg GR (2017). AMPK as a therapeutic target for treating metabolic diseases. Trends Endocrinol Metab.

[48] Liu CY, Zhou Y, Chen T, Lei JC, Jiang XJ (2020). AMPK/SIRT1 pathway is involved in arctigenin-mediated protective effects against myocardial ischemia-reperfusion injury. Front Pharmacol.

[49] Pfluger PT, Herranz D, Velasco-Miguel S, Serrano M, Tschop MH (2008). Sirt1 protects against high-fat diet-induced metabolic damage. Proc Natl Acad Sci U S A.

[50] Purushotham A, Schug TT, Xu Q, Surapureddi S, Guo X, Li X (2009). Hepatocyte-specific deletion of SIRT1 alters fatty acid metabolism and results in hepatic steatosis and inflammation. Cell Metab.

[51] Canto C, Gerhart-Hines Z, Feige JN, Lagouge M, Noriega L, Milne JC (2009). AMPK regulates energy expenditure by modulating NAD+ metabolism and SIRT1 activity. Nature.

[52] Yang Z, Kahn BB, Shi H, Xue BZ (2010). Macrophage alpha1 AMP-activated protein kinase (alpha1AMPK) antagonizes fatty acid-induced inflammation through SIRT1. J Biol Chem.

[53] Yoshizaki T, Schenk S, Imamura T, Babendure JL, Sonoda N, Bae EJ (2010). SIRT1 inhibits inflammatory pathways in macrophages and modulates insulin sensitivity. Am J Physiol Endocrinol Metab.

[54] Meng X, Tan J, Li M, Song S, Miao Y, Zhang Q (2017). Sirt1: role under the condition of ischemia/hypoxia. Cell Mol Neurobiol.

[55] Alves-Fernandes DK, Jasiulionis MG (2019). The role of SIRT1 on DNA damage response and epigenetic alterations in cancer. Int J Mol Sci.

[56] Li F, Zhang L, Xue H, Xuan J, Rong S, Wang K (2021). SIRT1 alleviates hepatic ischemia-reperfusion injury via the miR-182-mediated XBP1/NLRP3 pathway. Mol Ther Nucleic Acids.

[57] Rada P, Pardo V, Mobasher MA, Garcia-Martinez I, Ruiz L, Gonzalez-Rodriguez A (2018). SIRT1 controls acetaminophen hepatotoxicity by modulating inflammation and oxidative stress. Antioxid Redox Signal.

