# Supplementary material for: Notch-activated mesenchymal stromal/stem cells enhance the protective effect against acetaminophen-induced acute liver injury by activating AMPK/SIRT1 pathway
Source: Stem Cell Res Ther. 2022 Jul 16;13:318. doi: 10.1186/s13287-022-02999-6 (PMC9288678; doi:10.1186/s13287-022-02999-6)
Supplement: Supplementary file 1 — Additional file 1: Table S1. Primer sequences for the amplification. [file 13287_2022_2999_MOESM1_ESM.docx]

**Supplementary materials**

| **Supplementary Table 1: Primer sequences for the amplification** | | |
| --- | --- | --- |
| Target genes | Forward primers | Reverse primers |
| HPRT | 5’-TCAACGGGGGACATAAAAGT-3’ | 5’-TGCATTGTTTTACCAGTGTCAA-3’ |
| TNF-α | 5’- ACGGCATGGATCTCAAAGAC-3’ | 5’- AGATAGCAAATCGGCTGACG-3’ |
| IL-6 | 5’-CTCTGGGAAATCGTGGAAATG-3’ | 5’-AAGTGCATCATCGTTGTTCATACA-3’ |
| IL-1β | 5’-TGTAATGAAAGACGGCACACC-3’ | 5’-TCTTCTTTGGGTATTGCTTGG-3’ |
| MCP-1 | 5'-GAAGGAATGGGTCCAGACAT-3' | 5'-ACGGGTCAACTTCACATTCA-3' |
| CXCL1 | 5’-TGGCTGGGATTCACCTCAAGAACA-3’ | 5’-TTTCTGAACCAAGGGAGCTTCAGG-3’ |
| COX2 | 5’- GGTTGCTGGTGGTAGGAATGTTC-3’ | 5’- AAGACTGGTATTTCATCTGCCTGC-3’ |
| Notch1 | 5’- TGCCTGGACAAGATCAATGAG-3’ | 5’- CAGGTGTAAGTGTTGGGTCC-3’ |
| Notch2 | 5’- AGGCACCTGTATTGACCTTG-3’ | 5’- TCCAATCCTATCCATGCACTG-3’ |
| Notch3 | 5’- AGTTCACCTGTATCTGTATGGC-3’ | 5’- ATTGACTCGGTCCTTGCAG-3’ |
| Notch4 | 5’- CTGTGAGGGAAAGCTCCG-3’ | 5’- GGGCACATAAGTCCATCAGAG-3’ |
