# Supplementary figures and images for: Notch-activated mesenchymal stromal/stem cells enhance the protective effect against acetaminophen-induced acute liver injury by activating AMPK/SIRT1 pathway
Source: Stem Cell Res Ther. 2022 Jul 16;13:318. doi: 10.1186/s13287-022-02999-6 (PMC9288678; doi:10.1186/s13287-022-02999-6)

Suppl Fig. 1

Saline + MSC

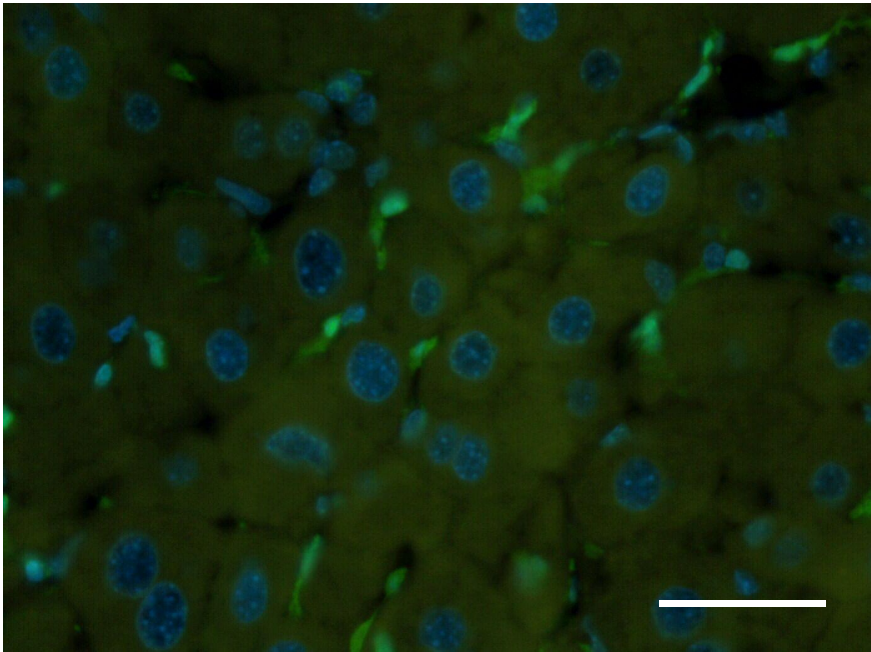

APAP + MSC

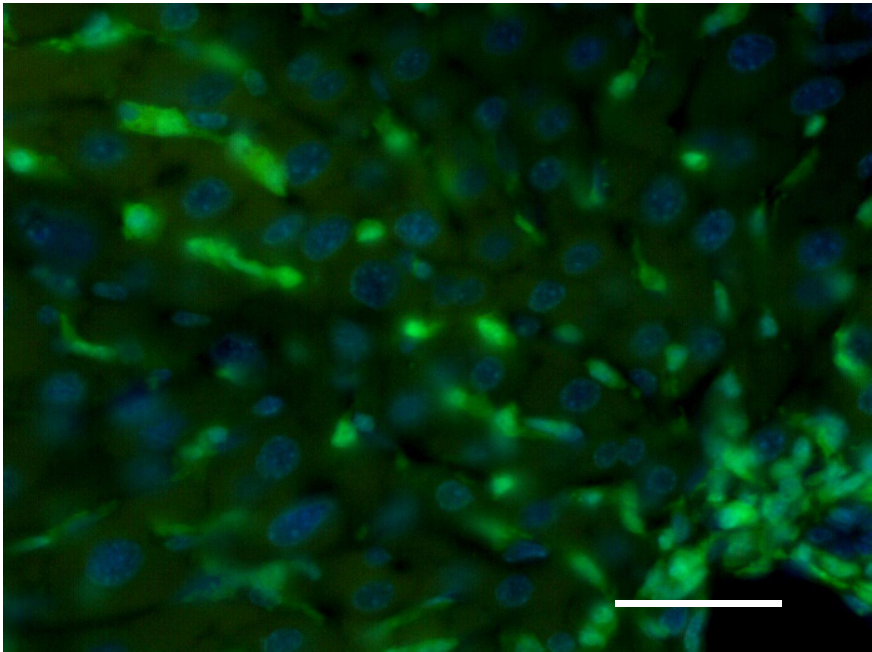

Supplement: Supplementary file 2 — Additional file 2: Fig. S1. Tracking of MSCs in the liver. Mice were injected through the tail vein with CellTrackerTM green CMFDA predyed MSCs (1X106) 24 h prior to APAP (400 mg/kg, i.p.) or saline injection. Representative immunofluorescence images for the MSCs labeled with CMFDA (green) localized in the liver. DAPI was used to visualize nuclei (blue). Scale bars: 20 μm. [file 13287_2022_2999_MOESM2_ESM.pdf]

Suppl Fig. 2

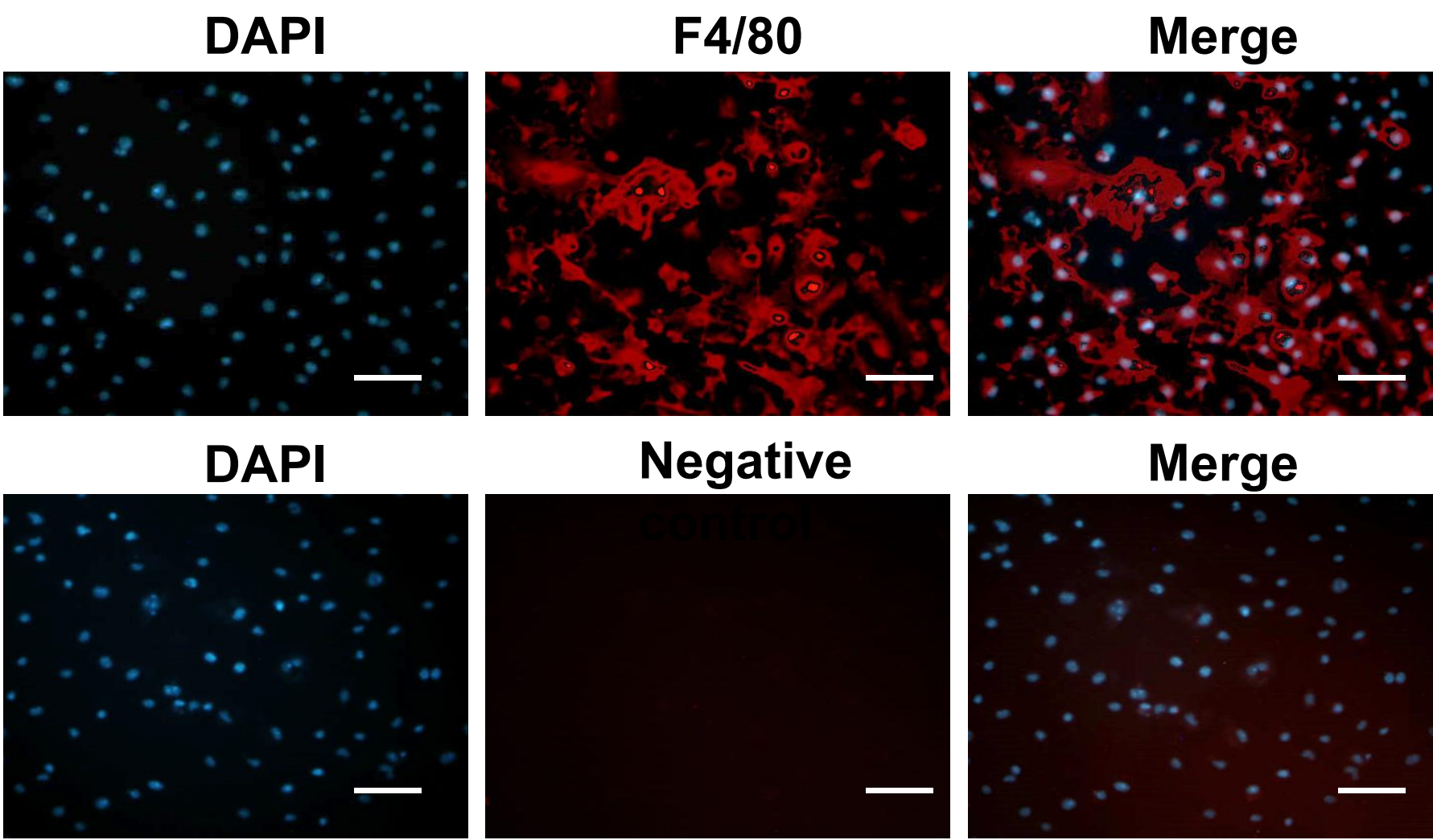

Supplement: Supplementary file 3 — Additional file 3: Fig. S2. Identification of isolated Kupffer cells. Hepatic macrophages (Kupffer cells) were isolated from mice. Representative immunofluorescence staining for F4/80 (red). DAPI was used to visualize nuclei (blue). Scale bars: 40 μm. [file 13287_2022_2999_MOESM3_ESM.pdf]

Suppl Fig. 3

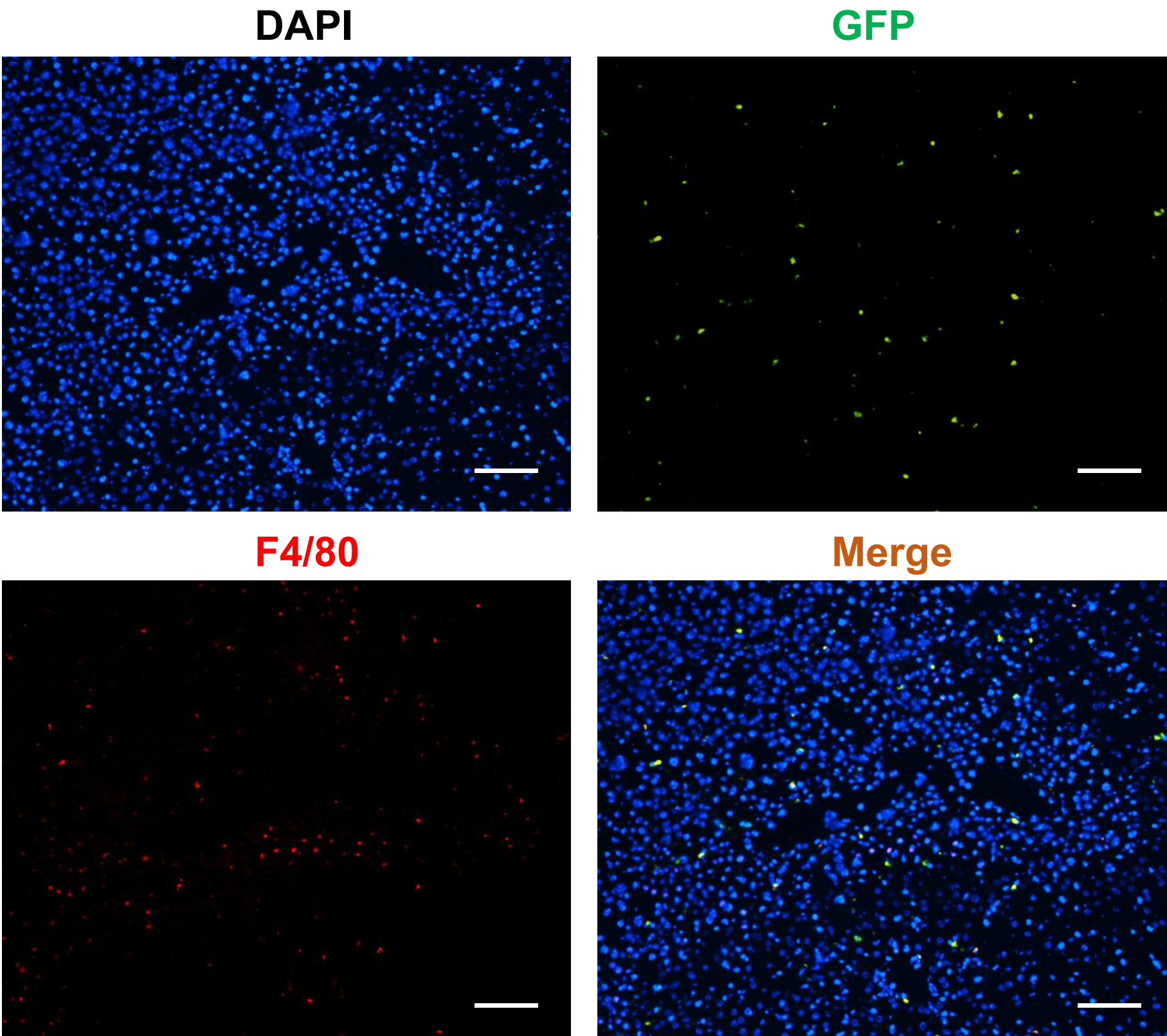

Supplement: Supplementary file 4 — Additional file 4: Fig. S3. Immunofluorescence staining of GFP-labeled and F4/80+ macrophages in liver tissue. Bone marrow-derived macrophages (BMDMs; 5×10^6^ cells/mouse) were transfected with lentivirus expressing GFP (Lv-GFP) and adoptively transferred into mice 24 h prior to APAP injection. DAPI was used to visualize nuclei (blue). Scale bars: 100 μm. [file 13287_2022_2999_MOESM4_ESM.pdf]
